# OUTCOMES OF EARLY SURGICAL REPAIR OF THE KNEE EXTENSOR MECHANISM INJURY, COMPARING THE QUADRICEPS AND PATELLAR TENDON INJURIES: A PROSPECTIVE OBSERVATIONAL STUDY

**DOI:** 10.1590/1413-785220253302e290211

**Published:** 2025-10-13

**Authors:** VINÍCIUS ANTÔNIO SANTOS ARAGÃO, JOÃO VICTOR LACERDA BELFORT, FLAVIO DE FAVA SANCHES, RENAN JOSÉ RIGONATO, MARIA ADELAIDE DE MIRANDA GONÇALVES, MARCOS DE CAMARGO LEONHARDT, JORGE DOS SANTOS SILVA, KODI EDSON KOJIMA

**Affiliations:** 1. Universidade de Sao Paulo, Faculdade de Medicina, Hospital das Clinicas (HCFMUSP), Instituto de Ortopedia e Traumatologia, Grupo de Trauma, Sao Paulo, SP, Brazil.

**Keywords:** Knee Injuries, Patellar Ligament, Quadriceps Muscle, Muscle Strength, Lysholm Knee Score, Traumatismos do Joelho, Ligamento Patelar, Músculo Quadríceps, Força Muscular, Escore de Lysholm para Joelho

## Abstract

**Objectives:**

this study aimed to compare the radiographic, functional and isokinetic results of the early repair of quadriceps tendon rupture (QTR) and patellar tendon rupture (PTR).

**Methods:**

We conducted a prospective observational study at a level one urban university trauma center from January 2022 to March 2023. The study included patients aged 18 years and older who underwent surgery within three weeks of injury and had at least one year of follow-up. The evaluation used the patellar indices (Insall-Salvati, Blackburn Peel and Caton Deschamps), functional (Lysholm and PCS-12) and isokinetic assessment.

**Results:**

the study involved 20 patients, predominantly male, with a slight predominance of left-sided injuries. The mean age was 39.4 years. Surgical outcomes showed no significant radiographic differences post-repair. Functionally, the PTR group exhibited better recovery compared to the QTR group, particularly in returning to sports and work. Isokinetic testing revealed a substantial reduction in strength on the injured side compared to the uninjured side across both groups.

**Conclusion:**

early surgical intervention leads to favorable radiographic and functional outcomes. The PTR group showed better functional recovery, especially in return to work and sports. No significant differences were found in isokinetic strength recovery between the QTR and PTR groups. Level of Evidence III; Prospective Observational Study.

## RESUMO

**Objetivo:**

este estudo teve como objetivo comparar os resultados radiográficos, funcionais e isocinéticos da reparação precoce da rutura do tendão do quadríceps (RQT) e da rutura do tendão patelar (RTP).

**Métodos:**

Realizamos um estudo observacional prospetivo em um centro de trauma universitário urbano de nível um de janeiro de 2022 a março de 2023. O estudo incluiu pacientes com 18 anos ou mais que foram submetidos à cirurgia dentro de três semanas após a lesão e tiveram pelo menos um ano de acompanhamento. A avaliação utilizou os índices patelares (Insall-Salvati, Blackburn Peel e Caton Deschamps), funcional (Lysholm e PCS-12) e avaliação isocinética.

**Resultados:**

o estudo envolveu 20 pacientes, predominantemente do sexo masculino, com ligeiro predomínio de lesões do lado esquerdo. A idade média foi de 39,4 anos. Os resultados cirúrgicos não mostraram diferenças radiográficas significativas após a reparação. Funcionalmente, o grupo RTP apresentou melhor recuperação em relação ao grupo RQT, nomeadamente no regresso ao desporto e ao trabalho. Os testes isocinéticos revelaram uma redução substancial da força no lado lesionado em comparação com o lado não lesionado em ambos os grupos.

**Conclusão:**

a intervenção cirúrgica precoce conduz a resultados radiográficos e funcionais favoráveis. O grupo PTR apresentou melhor recuperação funcional, especialmente no retorno ao trabalho e ao desporto. Não foram encontradas diferenças significativas na recuperação da força isocinética entre os grupos RQT e PTR. Nível de Evidência III; Estudo Observacional Prospectivo.

## INTRODUCTION

Injuries to the knee extensor mechanism significantly impact orthopedic practice, often leading to substantial morbidity, pain, and functional impairment.^1^ These injuries are categorized into three clinical types: quadriceps tendon rupture, patellar tendon rupture, and patellar fracture. Despite their differences, all types share a potential for knee extension deficit, often necessitating early surgical intervention.^2,3^

Quadriceps tendon ruptures (QTR) are approximately twice as common as patellar tendon ruptures (PTR), with incidences of 1.3% and 0.6%, respectively, among all soft tissue injuries in orthopedics.^4^ QTRs are frequently associated with tendinopathy, comorbidities, anabolic steroid use, and advanced age, typically occurring in patients over 40 years. Conversely, PTRs are more prevalent in younger patients (under 40 years), often resulting from repetitive microtrauma. The injury mechanisms to the knee extensor tendons can be direct or indirect, with indirect mechanisms—often due to eccentric contraction of the quadriceps muscle with the knee in flexion—being two to three times more common.^3-6^

Non-surgical management is appropriate for cases of partial tendon rupture where full knee extension is maintained. However, early surgical intervention within the first three weeks post-injury is recommended to prevent worse functional outcomes, often necessitating autologous graft reinforcement or reconstruction.^7-9^

Although postoperative outcomes are generally favorable, with a high rate of athletes returning to sports and high patient satisfaction, significant challenges remain. These include reductions in quadriceps muscle strength, loss of knee flexion, soft tissue shortening, and reports of residual pain.^8,10^

The primary aim of this study was to evaluate the functional and isokinetic outcomes of early surgical treatment for knee extensor mechanism tendon ruptures. The secondary outcome was to assess the radiographic outcomes of the tendon repairs.

## PATIENTS AND METHODS

This prospective observational study was conducted at a level one urban university trauma center from January 2022 to March 2023. The study received approval from the Research and Ethics Committee (approval number 81967024.4.0000.0068), and written informed consent was obtained from all participants.

The inclusion criteria were individuals aged 18 years and older, literate, with quadriceps or patellar tendon injuries, operated on within 3 weeks, with a minimum follow-up of one year, and who attended radiographic, functional, and isokinetic evaluations. Participants were also required to have signed an informed consent form.

The exclusion criteria included associated multiligamentar knee injury, ipsilateral patella fracture, previous knee fractures, associated ipsilateral injury, femoral or tibial nail fixation with entry point in the knee, and dementia syndrome.

The primary surgical method for tendon repair was transosseous reattachment of the tendon. The tendon end was exposed, nonviable tissue was removed, and a Krakow-type locking stitch suture was performed using a number five braided polyester non-absorbable suture (Ethibond, Ethicon, Somerville, NJ, USA). Three parallel longitudinal drill holes were made in the patella, and the four suture ends were passed through them. The tendon was anatomically reduced to the patella surface, and all sutures were tied, ensuring the patella was returned to its anatomical position in the trochlea, avoiding patella alta or baja. In cases with tear extension to the retinaculum, the injury was closed with number one Vicryl (Ethicon). At the end of the tendon repair, before final wound closure, the knee was moved from total extension to at least 110 degrees of flexion to ensure the quality of the repair.^11^

To reinforce quadriceps tendon repair, the Scuderi technique was used, involving a wide-based inverted V tendon flap. The tendon used to augment the primary repair of the patellar tendon was the ipsilateral autologous semitendinosus tendon, which was kept attached distally and looped around the proximal pole of the patella and sutured back to the patellar tendon distally.^11^

All patients used a knee brace in extension for two weeks, allowing weight-bearing as tolerated. Physiotherapy commenced in the third week.

Demographic data collected included age, sex, side of injury, BMI (Body Mass Index), comorbidities, smoking habits, previous symptoms in the knee, and sports activity. Treatment-related data included whether augmentation with autologous tendon was performed. Outcome-related data collected included re-rupture, return to work, and return to sport activity.

Radiographic measurements of the patellar indices (Caton-Deschamps, Blackburn-Peel, and Insall-Salvati) were performed using lateral knee radiographs taken with 30 degrees of flexion. The Caton-Deschamps index calculates the ratio between the patellar articular surface and its distal distance to the anterosuperior margin of the tibia. The Blackburn-Peel index compares the patellar articular surface with its distal distance to a line projected from the superior articular surface of the tibia. The Insall-Salvati index measures the ratio between the total length of the patella and the length of the patellar tendon. Normal values range between 1 and 1.2, while a ratio of 1.3 or greater indicates a high-riding patella.^7^

Functional outcomes were assessed using the Lysholm Knee Score questionnaire, and quality of life was evaluated using the Short Form (SF)-12v2, which provides mental and physical component summary scores (MCS-12 and PCS-12).^12-14^

The isokinetic test was conducted to assess the functional performance of the knee muscles, focusing on peak torque, peak torque to body weight ratio (peak BW), total work, and the hamstring-to-quadriceps (H/Q) ratio at two different angular velocities: 60 degrees/second and 240 degrees/second. Testing was performed with a Biodex System 3 dynamometer (Biodex Medical System, Inc., Shirley, NY). The 60 degrees/sec velocity was used to assess maximal strength, and the 240 degrees/sec velocity to evaluate muscle endurance.^15^

Qualitative characteristics were described using absolute and relative frequencies, while quantitative characteristics were summarized using descriptive statistics (mean and standard deviation). Parametric data were compared with the Student t-test, and the association between categorical variables with the Chi-square test of independence. Isokinetic measurements and functional scales were compared using paired Wilcoxon tests, and the correlation of percentage differences between the injured and contralateral sides with quantitative characteristics was examined using Spearman’s correlation. Differences in isokinetic measurements according to relevant qualitative characteristics were compared using Mann-Whitney tests. Data analysis was conducted using IBM-SPSS for Windows version 22.0, with tabulations in Microsoft Excel 2013, and a significance level set at 5%.^16^

## RESULTS

The study included 20 participants with a mean age of 39.4 years (median: 37 years). There were 8 patients (40%) in the group with QTR with a mean age of 46.7 ± 13.1 years and 12 patients (60%) in the PTR group with a mean age of 34.3 ± 13.5 years (p = 0.055). The cohort was predominantly male (95%) and primarily injured on the left side (70%). The mean Body Mass Index (BMI) was 28.2, indicating a tendency towards being slightly overweight. Most participants (70%) had no comorbidities, while 15% had one comorbidity, and another 15% had two or more. A logistic regression analysis was performed to examine the influence of comorbidities, diabetes, previous symptoms, smoking, and BMI on the variable local of injury to predict the value “0”. Logistic regression analysis showed that the model as a whole was not significant (Chi2(6) = 7.07, p = 0.315, n = 20). Regarding lifestyle factors, 20% of participants were smokers, and 65% engaged in regular sports activities (Table 1).

**Table 1:** Demographic results.

Variable	Total (N = 20)	QTR (N = 8)	PTR (N = 12)	p
Age (years) Mean ± SD	39.4 ± 14.5	46.9 ± 13.3	34.3 ± 13.5	0.055
Sex, n (%) Female Male	1 (5) 19 (95)	1 (12.5) 7 (87.5)	0 12 (100)	
Laterality, n (%) Right Left	6 (30) 14 (70)	2 (25) 6 (75)	4 (33.3) 8 (66.7)	
BMI Mean ± SD	28.2 ± 4.0	29.3 ± 5.0	27.5 ± 3.1	0.910
Comorbidities, n (%) None One Two or more	14 (70) 3 (15) 3 (15)	4 (50) 2 (25) 2 (25)	10 (83.4) 1 (8.3) 1 (8.3)	
Smoking, n (%) No Yes	16 (80) 4 (20)	6 (75) 2 (25)	10 (83.4) 2 (16.6)	
Previous symptoms, n (%) No Yes	13 (65) 7 (35)	6 (75) 2 (35)	7 (58.3) 5 (41.7)	
Sport activity, n (%) No Yes	7 (35) 13 (65)	5 (62.5) 3 (37.5)	2 (16.6) 10 (83.4)	
Tendon augmentation n(%) No Yes	12 (60) 8 (40)	5 (62.5) 3 (37.5)	7 (58.3) 5 (41.7)	

Student t test

Additionally, 40% of patients underwent autograft tendon augmentation during surgery—three in the QTR (37.5%) and five in the PTR (41.6%) (Table 1). Post-surgical radiographic scores showed no significant differences between sides. The Insall-Salvati Score for the injured knee had a mean of 1.3, compared to 1.27 for the healthy knee (p = 0.926). The Blackburn-Peel score was slightly higher in the injured knee, with a mean of 1.3 compared to 1.2 on the contralateral side (p = 0.422). The Caton-Deschamps score similarly showed no significant difference, with a mean of 1.52 for the injured knee compared to 1.54 on the contralateral side (p = 0.255) (Table 2).

**Table 2:** Radiographic patellar indices results.

Variable	QTR			PTR			
	Injured	Normal	p	Injured	Normal	p	p (Q/P)
Insall-Salvati	1.1 ± 0.1	1.2 ± 0.2	0.926	1.4 ± 0.4	1.3 ± 0.2	0.520	0.055
Blackburn Peel	1.0 ± 0.1	1.1 ± 0.3	0.422	1.4 ± 0.3	1.2 ± 0,2	0.120	0.070
Caton Deschamps	1.3 ± 0.3	1.5± 0.5	0.255	1.7 ± 0.3	1.6 ± 0.3	0.650	0.423

Paired Wilcoxon test. QTR = quadriceps tendon rupture, PTR = patellar tendon rupture.

Re-rupture occurred in 2 cases (10%)—one in each group—and reoperation was required in one (5%). In terms of recovery, 85% of participants returned to their previous work, and 69.2% of those previously active in sports returned to sports activities post-recovery. The statistical analyses of the correlation between return to work and the location of the injury showed a non-significant difference (p = 0.097). For the return to sports, the statistical analyses showed a marginal association of more return to sports in the PTR group than the QTR group, but it did not reach the threshold for statistical significance (p = 0.054) (Table 3).

**Table 3:** Functional results.

Variable	QTR (N = 8)	PTR (N = 12)	p
Re-rupture, n (%) No Yes	7 (87.5) 1 (12.5)	11 (91.7) 1 (8.3)	0.805
Return to work, n (%) No Yes	3 (37.5) 5 (62.5)	0 12 (100)	0.097
Return to sports, n (%) No Yes	7 (87.5) 1 (12.5)	4 (33.3) 8 (66.7)	0.054
Lysholm score Mean ± SD	63.5 ± 28.5	82.4 ± 15.8	0.114
PCS-12, n (%) Mean ± SD	1.4 ± 0.9	0.6 ± 0.5	0.045

Paired Wilcoxon test.

The Lysholm score for the PTR group (82.7 ± 15.8) was higher than the score for the QTR group (63.2 ± 28.5) (p = 0.114). The PCS-12 of the PTR group (0.6 ± 0.5) was significantly better than the QTR group (1.4 ± 0.9) (p = 0.045) (Table 3). In a 60° isokinetic evaluation, the injured knee exhibited a mean peak torque of 106.9 ± 58.4 Nm, while the uninjured knee showed a higher mean of 182.5 Nm (p < 0.001). The peak torque normalized to body weight (peak/BW) for the injured knee was 120.5 Nm/kg, compared to 203.2 Nm/kg for the uninjured knee (p < 0.001). Total work was also significantly higher on the uninjured knee, with a mean of 589.5 J compared to 345.1 J on the injured knee (p < 0.001). Similarly, the hamstring/quadriceps ratio (H/Q) at this level was higher on the injured knee, with a mean of 90.4, versus 56.7 on the uninjured knee (p < 0.001) (Table 4). At the 240° isokinetic evaluation, the injured knee showed a mean peak torque of 77.5 Nm, lower than the uninjured knee’s mean of 108.6 Nm (p = 0.002). The peak/BW ratio for the injured knee was 87 Nm/kg, compared to 126.8 Nm/kg for the uninjured knee (p < 0.001). The total work at this level was also significantly lower on the injured knee, with an average of 917.1 J, compared to 1332.8 J on the uninjured side (p < 0.001). The agonist/antagonist ratio for the injured knee was 88.4, while the uninjured knee had a mean of 63.8 (p < 0.001) (Table 4). No statistical differences were observed between quadriceps tendon and patellar ligament injuries in functional, radiographic, or isokinetic assessments. Surgical techniques with tendon augmentation did not show statistical differences across all functional, clinical, and radiographic assessments. There was also no difference between those who returned to their previous work activities and those who did not. Among those who returned to previous sports activities, only the difference in peak/BW and total work at 240° was statistically greater in those who resumed sports (p = 0.020 for both). The other characteristics evaluated did not statistically influence the percentage difference in isokinetic exams in this population (p > 0.05).

**Table 4:** Isokinetic assessment results.

Variable	QTR			PTR			
	Injury	Normal	p	Injury	Normal	p	p (Q/P)
60 peak torque	101.1 ± 50.3	168.9 ± 35.4	0.007	110.7 ± 65.1	191.5 ± 62.1	0.005	0.715
60 peak body weight	108.3 ± 55.6	181.7 ± 45.9	0.021	128.5 ± 76.7	217.5 ± 69.7	0.007	0.504
60 total work	320.4 ± 160.5	556 ± 118.1	0.004	361.5 ± 240.8	611.8 ± 211.8	0.013	0.652
60 H/Q	77.9 ± 26.0	53.4± 9.9	0.025	58.5 ± 14.4	98.8 ± 33.4	0.001	0.135
240 peak torque	64.5 ± 30	86 ± 34.5	0.205	86.1 ± 40.2	123.6 ± 32.7	0.020	0.187
240 peak body weight	69.4 ± 33.5	106.4 ± 23.9	0.023	98.7 ± 45.6	140.5 ± 37.4	0.022	0.116
240 total work	742.2 ± 419.6	1051 ± 307.6	0.115	1033.6 ± 524.8	1520.1 ± 442.5	0.022	0.187
240 H/Q	79.7 ± 23.4	64.1 ± 14.4	0.131	94.2 ± 48.7	63.5 ± 9.3	0.043	0.387

Paired Wilcoxon test. QTR = quadriceps tendon rupture, PTR = patellar tendon rupture.

## DISCUSSION

The present study aimed to compare the outcomes of acute repair of the knee extensor mechanism injury. Despite the limitation of a small sample size, the analysis focused on the differences between quadriceps tendon and patellar tendon injuries, comparing the demographics, clinical outcomes, radiographic, and functional assessments as described above.

Our results indicate a significant age difference between the two groups, with the mean age for patients with QTR being higher (46.9 years) compared to those with PTR (34.3 years, p = 0.017). Comparing to Garner et al.3 the mean age for PTR was similar (34.3 vs. 39.6 years), but for the QTR our patients showed a lower mean age (46.7 vs. 61.0 years). This may explain the absence of correlation of the QTR with comorbidities as described by Boudissa et al.^8^ where older patients tend to have more comorbidities.

The injury to the left knee represented 70% of the total, with a higher incidence in both the QTR (75%) and PTR (66.7%). We could not find in the literature neither a similar number nor an explanation for this higher incidence on the left side. This may be related to lower leg muscle dominance and protective responses.

The higher incidence of previous symptoms in the PTR (41.7%) compared to the QTR (25%) is consistent with the literature and shows the presence of degenerative changes predisposition to injury.^1,3^

The need for augmentation of the repair was at the surgeon’s discretion according to his impression of the amount of degeneration of the tendon and showed no difference in both groups (QTR 37.5% vs. PTR 41.6%). And the augmentation had no correlation with the radiographic and functional results (p > 0.05).

When the patient suffers a knee extensor mechanism injury, the position of the patella will depend on the rupture location; in the QTR, the patella tends to stay in its position or lower, whereas in the PTR the patella is usually in a higher position. This may lead to a repair with an altered patella position after the suture. But the radiographic measurements of the patellar indices (Caton-Deschamps, Blackburn-Peel, and Insall-Salvati) in our study showed that the repair reestablished the patellar height similar to the contralateral normal side in both the QTR and PTR, demonstrating the good reconstruction of the patellar height (Table 2).

In general, the patients with PTR had a better recovery compared to the patients with QTR, as assessed by return to previous work and sports activity. In the PTR group, all patients (100%) returned to their previous occupation, and 66.7% resumed sports activities. In contrast, only 37.5% of the patients with QTR returned to their previous work, and just 12.5% were able to return to sports. The differences could be explained by the greater mechanical demands placed on the quadriceps, as well as the older age and possibly lower baseline activity level of the QTR group.

The same happened in the objective assessment. The Lysholm score was higher in the PTR group (82.4 ± 28.5) compared to the QTR group (63.2 ± 12.5), indicating a better functional outcome. Similarly, the SF-12 physical component score was more favorable in the PTR group (0.6) versus the QTR group (1.4), with lower scores indicating better health. West et al.^17^ have also found a high Lysholm score ranging from 70 to 100, but they did not make a separate analysis between QTR and PTR. In their comparative study, Hantes et al.^18^ the Lysholm score had no difference between the groups (QTR 91 and PTR 85, p = 0.124). But they found better Kujala score and VAS in the QTR group.

The better functional result of the PTR group may be explained by the higher tensile strength and stiffness of the patellar tendon compared to the quadriceps tendon, which allows more effective force transmission during activities like jumping and running, and in contrast, the quadriceps tendon is more complex in its anatomy and its biomechanical function in the knee, leading to a less predictable healing.^19^

In the isokinetic evaluation, the peak torque and peak body weight ratio showed both groups with significant differences between the injured side and the normal side, with the injured side exhibiting 60% of the contralateral normal side, representing a substantial reduction of strength, affecting the knee’s ability to generate force during extension.

The total work also showed an endurance reduction of 40% on average in both QTR and PTR groups, indicating that the knee can do less work overtime in contrast to the normal side. The H/Q ratio just confirms the imbalance due to the quadriceps weakness.

The isokinetic results persisted at higher speed (240o), but less pronounced, suggesting that the deficit in muscle function in the injured knee is less detectable at higher speeds.

Comparing the isokinetic results of the injured knee between the QTR and PTR groups, the results showed similar levels of performance across all parameters (peak torque, peak BW ratio, total work, and H/Q ratio) and at different speeds (60o and 240o per second), with no statistically significant differences. This suggests functional equivalence between QTR and PTR groups in the injured knee, similar to the results of Yalcin et al.^20^

This study has some limitations. The study may have a small sample size, that can be explained by the low incidence of this injury, as shown by the number of patients included by Hantes et al. in their comparative study (n = 24) and Strother et al.^21^ in a ten-year study included only 43 patients. The sample size limits the comparability and generalizability of the results. The functional result may be influenced by variability of the rehabilitation by the patient regardless of the orientation given. The heterogeneity of the study population may introduce confounding variables. The use of self-reported measures, such as the Lysholm score and SF-12 physical component score, introduces a level of subjectivity. Patients’ perceptions of their recovery and function might be influenced by factors such as pain tolerance, psychological status, or expectations, which could lead to variability in the results.

We can conclude that the PTR occurs in younger patients, more on the left side, with more previous symptoms, have better Lysholm and SF-12-PS scores, and similar isokinetic assessment compared to the QTR group. In both groups, all three patellar indices showed similar results in comparison to the normal side.

## CONCLUSIONS

The Lysholm and SF-12 PS scores were better in the PTR group. All isokinetic measurements showed similar results between the PTR and QTR groups, with an average 40% deficit of the operated knee. The radiographic indices showed similar ratios between the operated and normal knee.
